# Growth differentiation factor 11 delivered by dairy *Lactococcus lactis* strains modulates inflammation and prevents mucosal damage in a mice model of intestinal mucositis

**DOI:** 10.3389/fmicb.2023.1157544

**Published:** 2023-04-17

**Authors:** Monique Ferrary Américo, Andria dos Santos Freitas, Tales Fernando da Silva, Luís Cláudio Lima de Jesus, Fernanda Alvarenga Lima Barroso, Gabriela Munis Campos, Rhayane Cristina Viegas Santos, Gabriel Camargos Gomes, Rafael Assis, Ênio Ferreira, Pamela Mancha-Agresti, Juliana Guimarães Laguna, Jean-Marc Chatel, Rodrigo Dias de Oliveira Carvalho, Vasco Azevedo

**Affiliations:** ^1^Department of Genetics, Ecology, and Evolution, Institute of Biological Sciences, Federal University of Minas Gerais, Belo Horizonte, Brazil; ^2^INRAE, Institut Agro Rennes-Angers, STLO, Rennes, France; ^3^Department of General Pathology, Federal University of Minas Gerais, Belo Horizonte, Brazil; ^4^Federal Center for Technological Education of Minas Gerais, Belo Horizonte, Brazil; ^5^INRAE, AgroParisTech, MICALIS, Université Paris-Saclay, Jouy-en-Josas, France; ^6^Department of Biochemistry and Biophysics, Institute of Health Sciences, Federal University of Bahia, Salvador, Brazil

**Keywords:** intestinal inflammation, 5-fluorouracil, lactic acid bacteria, DNA delivery, gene therapy

## Abstract

Mucositis is an inflammation of the gastrointestinal mucosa that debilitate the quality of life of patients undergoing chemotherapy treatments. In this context, antineoplastic drugs, such as 5-fluorouracil, provokes ulcerations in the intestinal mucosa that lead to the secretion of pro-inflammatory cytokines by activating the NF-κB pathway. Alternative approaches to treat the disease using probiotic strains show promising results, and thereafter, treatments that target the site of inflammation could be further explored. Recently, studies reported that the protein GDF11 has an anti-inflammatory role in several diseases, including *in vitro* and *in vivo* results in different experimental models. Hence, this study evaluated the anti-inflammatory effect of GDF11 delivered by *Lactococcus lactis* strains NCDO2118 and MG1363 in a murine model of intestinal mucositis induced by 5-FU. Our results showed that mice treated with the recombinant lactococci strains presented improved histopathological scores of intestinal damage and a reduction of goblet cell degeneration in the mucosa. It was also observed a significant reduction of neutrophil infiltration in the tissue in comparison to positive control group. Moreover, we observed immunomodulation of inflammatory markers *Nfkb1, Nlrp3, Tnf*, and upregulation of *Il10* in mRNA expression levels in groups treated with recombinant strains that help to partially explain the ameliorative effect in the mucosa. Therefore, the results found in this study suggest that the use of recombinant *L. lactis* (pExu:*gdf11*) could offer a potential gene therapy for intestinal mucositis induced by 5-FU.

## Introduction

Mucositis is manifested as an inflammation of the gastrointestinal tract (GIT) as a side effect caused by radio and chemotherapy treatment of cancers. The disease has been reported either in the upper (oral mucositis) or lower GIT (intestinal mucositis) in 50−80% of patients using the anti-metabolite drug 5-fluorouracil (5-FU) (Dahlgren et al., 2021; Sougiannis et al., 2021). Clinically, mucositis patients present symptoms like dysphagia, nausea, intense pain, bleeding, and diarrhea, which are crucial limiting factors for anti-neoplastic treatment continuity (Sonis et al., 2004; Batista et al., 2020). 5-FU-triggered ulcerations are caused by the inhibition of cell proliferation in both malignant and healthy tissue through blockage of nucleic acid synthesis, which consequently leads to apoptosis and generation of Reactive Oxygen Species (ROS). The disruption of the epithelial barrier activates the NF-κB pathway, leading to the production of the pro-inflammatory cytokines TNF, IL-1β and IL-6 and tissue infiltration by immune cells such as neutrophils, which contribute to inflammatory signal amplification (Villa and Sonis, 2015; Carvalho et al., 2018). This cascade of events culminates in the destruction of the intestinal architecture with crypt and villi loss, leaving the GIT vulnerable to potential infection by opportunistic bacteria due to mucosal destruction and altered intestinal permeability (Li et al., 2017). To date, no effective intervention is available to treat or prevent mucositis (Dahlgren et al., 2021).

Pre-clinical experimental models have reported promising results with the use of probiotics as alternative therapeutic approaches for mucositis (de Jesus et al., 2019) and other intestinal inflammation disorders, since these microorganisms can maintain epithelial barrier integrity (Blackwood et al., 2017), regulate intestinal dysbiosis (Shi et al., 2017), induce mucin production, and produce bacteriocins and antimicrobial substances such as lactic acid to inhibit Gram-negative bacteria (Castilho et al., 2019), among other mechanisms. Despite the positive effects reported, it is necessary to screen strains, adjust dose and length of treatment and recently, some studies suggest that a combination of treatments that target different aspects of GIT inflammation could provide more efficient protection (Quaresma et al., 2019; Dahlgren et al., 2021). In this context, using genetically modified strains with probiotic properties to express or deliver therapeutic molecules emerge as an attractive alternative to treat inflammatory diseases. Studies report that *Lactococcus lactis*, the model lactic acid bacteria, demonstrated an amelioration effect on intestinal damage in mice models of Ulcerative Colitis (UC) when modified to secrete 15-lipoxygenase and deliver IL-4 as a gene therapy (Carvalho, 2016; Souza et al., 2016). However, unlike other inflammatory models, few studies explore the use of recombinant microorganisms in experimental models of mucositis (Caluwaerts et al., 2010; Carvalho et al., 2017a; Barroso et al., 2021), remaining a field that must be more explored and therefore, it opens perspectives to search for new anti-inflammatory molecules for developing therapeutic and preventive approaches for mucositis and other inflammatory conditions.

In this context, recent scientific studies have demonstrated the anti-inflammatory effects of Growth Differentiation Factor 11 (GDF11) in different experimental models of inflammatory diseases such as arthritis, psoriasis, and Ulcerative Colitis (Wang et al., 2018, 2019; Li et al., 2019). GDF11 belongs to the TGF-β superfamily and its anti-inflammatory role seems to be mainly associated with the suppression of the NF-κB pathway, involved in the activation of TGF-β/Smad2/3 pathway while suppressing NF-κB and JNK pathways (Mei et al., 2016; Zhang et al., 2016). However, other non-canonical signalization pathways have been reported to be activated by GDF11, such as the MAPK pathway (Xia et al., 2019). Moreover, beneficial effects have been reported for GDF11. Recombinant GDF11 gene transfer (AAV-GDF11) promoted the protection of injured endothelium, attenuated apoptosis, and reduced expression of innflammatory markers *Tnf, Il1b, Mcp1, Il6* in an experimental atherosclerosis model in mice (Mei et al., 2016). An amelioration was also observed in mice following the administration of the rGDF11 protein in a rheumatoid arthritis model, alleviating the arthritis phenotype that could be associated with the inhibition of the NF-κB pathway and downregulation of pro-inflammatory cytokines *Tnf, Il1b*, and *Il6* expression (Li et al., 2019). Furthermore, human rGDF11 administration in mice attenuated colitis induced by DSS, and this effect was mainly attributed to the regulation of inflammasome complex activation by reducing the expression of TLR4/NF-κB, IL6 and IL1β (Wang et al., 2018). Taken together, these pieces of evidence highlight the potential of GDF11 as a protein with anti-inflammatory properties to be explored in other experimental models, such as intestinal mucositis.

Hence, based on recent studies reporting the positive effects of the anti-inflammatory role of GDF11 in different experimental models and current evidence regarding recombinant *L. lactis* as a delivery vehicle in IBDs, this study evaluated the effects of rGDF11 delivered by *L. lactis* strains to ameliorate the 5-FU-induced intestinal mucositis in mice.

## Materials and methods

### Bacterial strains, growth conditions, and plasmids

*Escherichia coli* TOP10 (Invitrogen, Carlsbad, CA, USA) was grown in Luria-Bertani (LB) medium (1% peptone, 0.5% NaCl, 0.5% yeast extract) supplemented with erythromycin (125 μg/mL) or ampicillin (100 μg/mL) when necessary, at 37°C, under agitation (150 rpm) for 16 h. Recombinant *E. coli* strains were stocked in glycerol solution (80%, 1:1) at −80°C until further use. *Lactococcus lactis* strains were grown in M17 (Sigma-Aldrich, San Luis, MI, USA) supplemented with glucose (0.5%, GM17) and supplemented with erythromycin (125 μg/mL), when necessary, for growth of recombinant strains harboring pExu plasmid at 30°C overnight. Recombinant strains were stocked in glycerol solution (80%, 1:4) at −80°C until use. All strains and plasmids utilized in this study are listed in Table 1.

**TABLE 1:** Characteristics of strains and plasmids utilized in this study.

Strain	Characteristics	Source
*Escherichia coli* TOP10	F^–^ (hsdR, mcrA, laczΔM15, endA1, recA1)	Invitrogen (Carlsbad, CA, USA)
*Escherichia coli* TOP10 pCloneEZ-NRS-Blunt-Amp:*gdf11*	(*E. coli* TOP10 carrying pCloneEZ-NRS-Blunt-Amp:*gdf11*, amp^r^)	This work
*Escherichia coli* TOP10 pExu:*gdf11*	(*E. coli* TOP10 harboring plasmid pExu:*gdf11*, ery^r^)	This work
*Lactococcus lactis* subsp. *cremoris* MG1363	Wild type strain, free of plasmids	Gasson, 1983
*Lactococcus lactis* subsp. *cremoris* MG1363 pExu (empty)	(*L. lactis* subsp. *cremoris* MG1363 harboring pExu empty vector, ery^r^)	Mancha-Agresti et al., 2017
*Lactococcus lactis* subsp. *cremoris* MG1363 pExu:*gdf11*	(*L. lactis* subsp. *cremoris* MG1363 harboring pExu:*gdf11*, ery^r^)	This work
*Lactococcus lactis* subsp. *lactis* NCDO 2118	Wild type strain, free of plasmids	Miyoshi et al., 2004
*Lactococcus lactis* subsp. *lactis* NCDO 2118 pExu (empty)	(*Lactococcus lactis subsp. lactis* NCDO 2118 harboring pExu empty vector, ery^r^)	Laboratory of Cellular and Molecular Genetics Collection
*Lactococcus lactis* subsp. *lactis* NCDO 2118 pExu:*gdf11*	(*Lactococcus lactis* subsp. *lactis* NCDO2118 harboring pExu empty vector, ery^r^)	This work
**Plasmids**	**Characteristics**	**Source**
pCloneEZ-NRS-Blunt-Amp:*gdf11*	(ori, pcat, ampR, *gdf11*)	GenOne (Rio de Janeiro, RJ, Brazil)
pExu (empty)	(pCMV, repD, repE, ery^r^)	Mancha-Agresti et al., 2017
pExu:*gdf11*	(pCMV, repD, repE, ery^r^, *gdf11*)	This work

F-, negative for plasmid F; hsdR, host restriction endonuclease R; mcrA, 5-methylcytosine-specific restriction enzyme; lacZΔM15, partial deletion of gene lacZ β-galactosidase; endA1, endonuclease A1; recA1, recombinase A1; ery^r^, erythromycin resistance; ori, replication origin; pcat, cat promoter; amp^r^, ampicillin resistance; gdf11, growth differentiation factor 11; pCMV, cytomegalovirus promoter; repD, replication protein D; repE, replication protein E.

### Construction of recombinant *Lactococcus lactis* (pExu:gdf11)

All DNA manipulations were performed following Sambrook and Russel (2001) protocols. To construct the eukaryotic expression vector pExu:*gdf11* the coding sequence of murine *gdf11* (GenBank accession number: NM_010272.2) was acquired in commercial plasmid pCloneEZ-NRS-Blunt-Amp:*gdf11*, and digested with restriction enzymes *Bam*HI (10 units) and *Not*I (20 units) for obtaining a 1,241 bp DNA fragment. Simultaneously, the plasmid pExu was digested with the same enzymes. DNA insert and vector were purified after running an agarose gel electrophoresis using Illustra*™* GFXTM PCR DNA kit (GE Healthcare, Chicago, IL, USA) following manufacturer instructions, and the molecular ligation step was performed with T4 DNA ligase (Invitrogen, Carlsbad, CA, USA) for 16 h at 4°C. The recombinant plasmid obtained was transformed into *E. coli* TOP10 by electroporation of competent cells (2,500 V, 200 Ω resistance, 25 μF capacitance pulse in a 2 mm cuvette) utilizing Eporator (Eppendorf, Hamburg, Germany). To select positive colonies, transformed cells were plated on LB agar medium supplemented with erythromycin overnight at 37°C. Purified pExu:*gdf11* DNA was isolated from cells utilizing the commercial kit Wizard Plus Minipreps DNA Purification System (Promega, Madison, WI, USA). Confirmation of pExu:*gdf11* construction was performed by double restriction enzyme digestion (*Bam*HI/*Not*I), previously described, and resolved into agarose gel electrophoresis.

To construct recombinant *Lactococcus lactis* strains MG1363 and NCDO2118 carrying pExu:*gdf11*, 1 μg of purified plasmid was transformed into competent wild-type cells by electroporation (2,500 V, 200 Ω resistance, 25 μF capacitance pulse in a 2 mm cuvette) using Eporator (Eppendorf, Hamburg, Germany) and transformed cells were plated on agar GM17 medium supplemented with erythromycin. Positive colonies were isolated by growing on liquid GM17 medium supplemented with erythromycin for 48 h at 30°C, and confirmation of the recombinant plasmid was performed by plasmid extraction resolved into electrophoresis as previously described above.

### Mice handling and experimental design

Four weeks old BALB/c male mice were provided by Biotério Central animal facility at the Federal University of Minas Gerais (Belo Horizonte, MG, Brazil). Animals were kept in polycarbonate-ventilated cages under controlled conditions: 25 ± 2°C, 12 h light/dark cycle, 55 ± 10% humidity, and were provided water and a standard chow diet *ad libitum* until the start of the experiment. All procedures were done under the National Council of Animal Experimentation (CONCEA) guidelines and approved by the Animal Experimentation Ethics Committee (CEUA-UFMG, protocol 122/2021, 28 June 2021). Mice were divided randomly into six experimental groups (*n* = 6 animals per group): negative/naive control (NEG); positive mucositis control (MUC); *L. lactis* strain MG1363 (pExu:empty) [MG1363 pExu (empty)]; *L. lactis* strain NCDO2118 (pExu:empty) [NCDO pExu (empty)]; *L. lactis* strain MG1363 (pExu:*gdf11*) (MG1363 pExu:*gdf11*) and *L. lactis* strain NCDO2118 (pExu:*gdf11*) (NCDO pExu:*gdf11*) treatment groups. Regarding the treatment rationale, mice received 10^9^ CFU daily of recombinant strains or Phosphate Saline Buffer (PBS, pH 7,4) via gavage for 13 days. During this period, body weight was assessed once a day. On the 10th day, mice were given an intraperitoneal injection of 5-fluorouracil (300 mg/kg, Flusan^®^, Eurofarma, São Paulo, SP, Brazil). The negative control received an injection of PBS. 72 h after mucositis induction, all animals were anesthetized with a ketamine (80 mg/kg) and xylazine (16 mg/kg) mixture solution and euthanized by cervical dislocation. The ileum section of the small intestine was collected for further analysis.

### Histological and morphometrical analysis and goblet cell count

Histological analysis was employed to evaluate the damage to the intestinal mucosa. The ileum section was collected after euthanasia and washed with PBS 0.1 M, rolled up, placed into a histological cassette, and immersed in 10% neutral buffered formalin solution (10% formaldehyde 40%, 0.4% NaH_2_PO_4_, 0.65% Na_2_H_2_PO_4_) for tissue fixation. The tissue was embedded in paraffin, and 4 μm-thick slices of samples were placed on a glass slide and stained with hematoxylin and eosin (HE) or periodic acid–Schiff (PAS). To evaluate the intestinal damage, the histological score proposed by Howarth et al. (1996) was applied, ranging from 0 (normal) to 3 (maximum damage) to the following parameters: a) villus fusion and stunting (atrophy); b) disruption of brush border and surface enterocytes; c) crypts loss/architectural disruption; d) infiltration of polymorphonuclear cells and lymphocytes; e) edema and thickening of the submucosal and muscular external layers (Howarth et al., 1996). Results were presented as a total sum of all parameters per animal per group. Posteriorly, image acquisition was performed with a 20x magnification objective using Olympus BX41 (Tokyo, Japan) to measure 20 villi and 20 crypts randomly selected per animal per group to measure crypt depth and villi height. Images were analyzed using ImageJ software (NIH, Bethesda, MD, USA). The assessment of goblet cells was performed in PAS-stained slides, where 10 field/slides were counted with a 40x magnification objective utilizing ImageJ software, and results were presented as average cells per field per group of animals.

### Myeloperoxidase activity assay (MPO)

The MPO enzyme activity properly evaluates the neutrophil tissue infiltration. Briefly, 100 mg of ileum was homogenized in 1.9 mL of Buffer 1 (NaCl 0.1 M; Na_2_EDTA 0.015 M, pH 4.7, 4°C) and metal beads (pH 7,4) using tissue homogenizer Precellys24^®^ (Bertin Instruments, Montigny-le-Bretonneux, France). The homogenate was centrifuged (10,000 rpm, 4°, 10 min), and NaCl 0.2% was added to the pellet followed by the addition of 1.9 mL of NaCl 1.6% supplemented with glucose 5%. Samples were centrifuged (10,000 rpm, 4°, 10 min), and Buffer 2 [NaH_2_PO_4_; 0.5% hexadecyltrimethylammonium bromide (HTAB, Sigma-Aldrich, St. Louis, MO, USA), pH 5.4], was added to the reminiscent pellet. The tissue suspension was homogenized in microtubes, submitted to three freeze-thawing cycles in liquid nitrogen, and then centrifuged for 10 min (10,000 rpm, 4°C). The colorimetric assay was performed by adding 25 μL of each sample to 25 μL of 1.6 mM 3,3,5,5′-tetramethylbenzidine (TMB, Sigma-Aldrich, St. Louis, MO, USA) in dimethyl sulfoxide (DMSO, Sigma-Aldrich, St. Louis, MO, USA) to a 96-well plate (Kasvi, São Paulo, SP, Brazil). Then, 100 μL of H_2_O_2_ 0.02% was added to the reaction and incubated at 37°C for 5 min. The reaction was stopped by adding 50 μL of H_2_SO_4_ 1 M. Absorbance was measured at 450 nm on a microplate reader (Bio-Rad 450 model, Bio-Rad Laboratories, Hercules, CA, USA), and results were expressed as arbitrary units per milligram of tissue based on absorbance.

### RNA extraction and real-time qPCR analysis

To evaluate gene expression of GDF11 cDNA delivered and inflammatory markers, 1 cm of ileum tissue previously stored in RNAlater^®^ reagent (Invitrogen, Carlsbad, CA, USA) was submitted to total RNA extraction using commercial PureLink™ RNA Mini Kit (Invitrogen, Carlsbad, CA, USA) following manufacturer instructions. RNA purity and concentration were assessed using NanoDrop^®^ 2000 (Thermo Scientific, Waltham, MA, USA) spectrophotometer considering absorbance ratios of 280/260 nm and 260/230 nm and was posteriorly evaluated in an agarose electrophoresis gel. The RNA extracted was treated with DNAse I (Invitrogen, Carlsbad, CA, USA) for 15 min at room temperature, followed by inactivation at 65°C for 10 min in EDTA 25 mM. cDNA synthesis was performed with 1 μg of RNA using a High-Capacity cDNA Reverse Transcription kit (Thermo Fisher Scientific, Waltham, MA, USA) following manufacturer instructions. The cDNA concentrations were evaluated using NanoDrop 2000 (Thermo Scientific, Waltham, MA, USA). Quantitative real-time PCR was performed using 200 ng of cDNA samples using Applied Biosystems SYBR Green Master Mix (Thermo Fisher Scientific, Waltham, MA, USA). Specific primers for genes *Gdf11*, *Actb*, *Gapdh*, *Il10*, *Nfkb1*, *Nlrp3*, and *Tnf* are listed in Table 2. The expression levels were presented as fold change using the 2^–ΔΔCT^ method.

**TABLE 2:** Oligonucleotides sequences utilized in this study for evaluation of relative gene expression.

Gene	Sequence	References
*Actb*	Fw: GCTGAGAGGGAAATCGTGCGTG Rv: CCAGGGAGGAAGAGGATGCGG	Volynets et al., 2016
*Gapdh*	Fw: TCACCACCATGGAGAAGGC Rv: GCTAAGCAGTTGGTGGTGCA	Giulietti et al., 2001
*Gdf11*	Fw: ATCAGCCGGGAGGTAGTGAA Rv: CTGGGCCATGCTTATGACCGT	Dichmann et al., 2006
*Il10*	Fw: GGTTGCCAAGCCTTATCGGA Rv: ACCTGCTCCACTGCCTTGCT	Giulietti et al., 2001
*Nfkb1* (p105)	Fw: GTGGAGGCATGTTCGGTAGTG Rv: TCTTGGCACAATCTTTAGGGC	Zheng et al., 2017
*Nlrp3*	Fw: AGAGCCTACAGTTGGGTGAAATG Rv: CCACGCCTACCAGGAAATCTC	Chen et al., 2019
*Tnf*	Fw: CATCTTCTCAAAATTCGAGTGACAA Rv: TGGGAGTAGACAAGGTACAACCC	Sakai et al., 2013

### Statistical analysis

All statistical analyses were performed with GraphPad Prism 8.0 software. Data normality was assessed utilizing the Shapiro–Wilk test. Non-parametric data were submitted to Kruskal-Wallis analysis and Dunn’s *post-hoc* test. Standard distribution data were submitted to one-way ANOVA following Tukey’s post-test. Statistical differences were considered using a *p*-value < 0.05.

## Results

### Recombinant *L. lactis* (pExu:*gdf11*) did not prevent weight loss in 5-FU-treated mice

**FIGURE 1:**
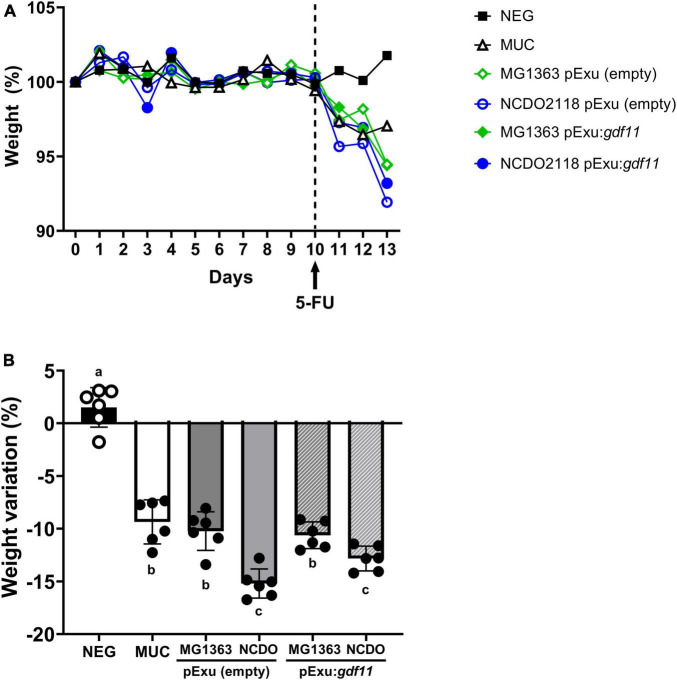
Evaluation of beneficial effect of recombinant *L. lactis* (pExu:*gdf11*) on body weight of mice (*n* = 6) inflamed with 5-FU. **(A)** Daily average body weight of mice treated during 13 experimental days. **(B)** Detailed average body weight loss observed after mucositis induction with 5-FU shows a significant decrease compared to the negative group. Different letters (a, b, and c) represent the significant difference (*p* < 0.05) among groups by one-way ANOVA and Tukey’s post-test.

The body weight of animals was assessed daily (Figure 1A) to evaluate the ability of recombinant strains to prevent weight loss, a clinical feature of intestinal mucositis. Body weight measured on days 10 to 13 decreased significantly (*p* < 0,0001) in mice that received 5-FU, and treatment with recombinant strains *L. lactis* (pExu:*gdf11*) did not prevent weight loss (Figure 1B). No mortality or abnormal clinical behavior was observed during the experiment.

### Recombinant *L. lactis* NCDO2118 (pExu:*gdf11*) prevented mucosal damage and goblet cell degeneration

**FIGURE 2:**
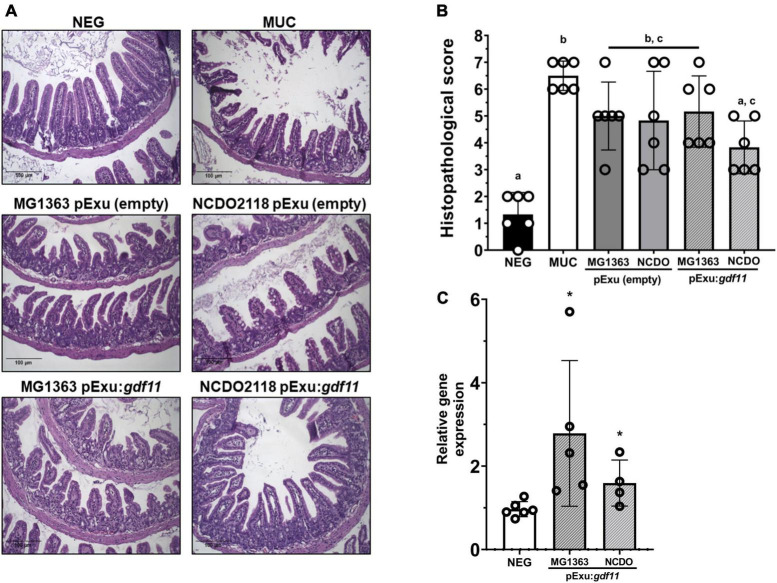
Protective effect of recombinant *L. lactis* (pExu:*gdf11*) on histopathological mucosa damaged by 5-FU and *Gdf11* expression. **(A)** Representative photomicrographs of experimental groups captured in 20X objective, scale 100 μm. **(B)** Histopathological score (*n* = 6) of mucosal damage caused by 5-FU. Median of the total score. Different letters (a, b, and c) indicate a significant difference (*p* < 0.05) among groups through Kruskal–Wallis analysis, followed by Dunn’s multiple comparison test. **(C)** Relative expression of *Gdf11* in the ileum of mice (*n* = 4–6) following 2^ΔΔCt^ method of analysis. Statistical significance was indicated by *(*p* < 0.05) followed by Student’s *t*-test.

The histopathological investigation is essential for assessing the severity of mucosal damage led by 5-FU. Figure 2A shows representative images of the ileum intestinal mucosa from experimental groups. As expected, tissue integrity was found well preserved in the negative group (Figure 2A, NEG). Mice that received 5-FU presented atrophy of villus, crypt loss, and elevated presence of polymorphonuclear inflammatory cells (Figure 2A, MUC). Lower mucosal damage was observed only in the group that received *L. lactis* strain NCDO2118 (pExu:*gdf11*). This group presented preservation of intestinal architecture and maintenance of the brush border and surface of enterocytes, thus, presenting a significant decrease in the histopathological score when compared to the MUC group (*p* < 0.05) (Figure 2B). This effect was not observed in the group that received recombinant *L. lactis* MG1363 (pExu:*gdf11*) and the bacterial control groups.

The *Gdf11* expression levels were assessed to confirm the gene therapy delivery. Results showed higher expression of *gdf11* compared to the negative control group (Figure 2C), hence, the administration of recombinant *L. lactis* strains successfully delivered GDF11 as a gene therapy directly on the damaged intestinal mucosa.

**FIGURE 3:**
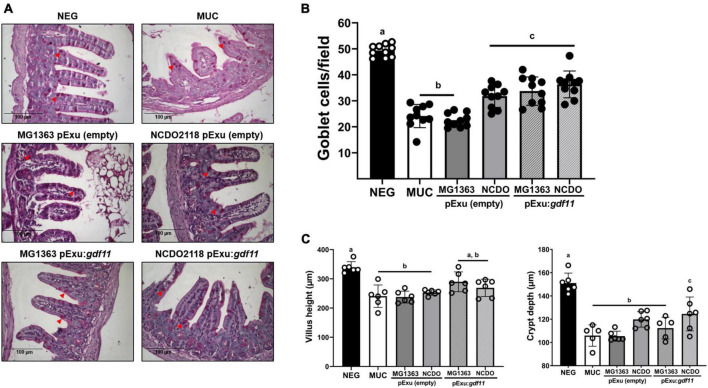
Protective effect of recombinant *L. lactis* (pExu:*gdf11*) on goblet cells number in the intestinal mucosa and average villus height (μm) and crypt depth of mice that received 5-FU for induction of intestinal mucositis. **(A)** Representative photomicrographs from the ileum section stained with Periodic-acid-Schiff (PAS). Red arrowheads indicate intact goblet cells in the tissue (40x objective, 100 μm scale). **(B)** Average number of goblet cells per field encountered in experimental groups (*n* = 6). Different letters (a, b, and c) indicate a significant difference (*p* < 0.05) among groups by one-way ANOVA and Tukey’s post-test. **(C)** Mice (*n* = 6) presented villus shortening and crypt depth reduction as a consequence of 5-FU administration. Different letters (a, b, and c) indicate a significant difference (*p* < 0.05) among groups by one-way ANOVA and Tukey’s post-test.

A significant decrease in goblet cells in the intestine of animals was observed in the control groups (MUC and mice that received strain MG1363 harboring empty vector) (Figure 3B) compared to the negative group. A significant increase in goblet cells was observed in groups that received strain NCDO2118 harboring empty vector and this effect was also observed in recombinant *L. lactis* (pExu:*gdf11*) groups, demonstrating that the administration of these strains was able to hinder goblet cell degeneration in the section of intestinal mucosa evaluated. Villus height and crypt depth measurement showed that all groups that received 5-FU presented villus shortening and crypt depth reduction as a consequence of mucosal damage compared to the negative control (Figure 3B). Treatment with recombinant strains *L. lactis* (pExu:*gdf11*) did not ameliorate these parameters.

### Recombinant *L. lactis* NCDO2118 (pExu:*gdf11*) reduced neutrophils infiltrate

**FIGURE 4:**
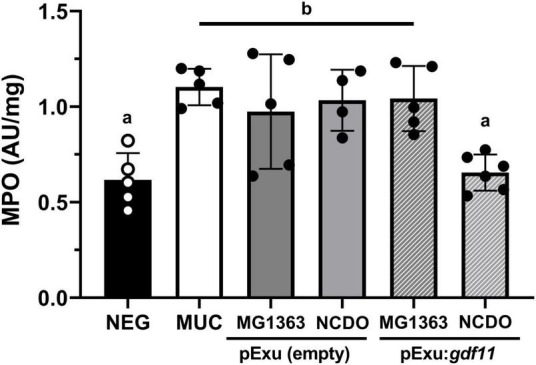
Effect of recombinant *L. lactis* (pExu:*gdf11*) on myeloperoxidase activity (MPO) on the ileum of mice (*n* = 5). Different letters (a and b) indicate a significant difference (*p* < 0.05) among groups by one-way ANOVA and Tukey’s post-test.

We performed an MPO activity assay to evaluate the presence of infiltrating neutrophils (Figure 4). Results showed the presence of infiltrating cells in groups that received 5-FU for mucositis induction; however, mice receiving recombinant *L. lactis* NCDO2118 (pExu:*gdf11*) showed a significant decrease (*p* < 0.01) in MPO activity, therefore supporting the histology results. No beneficial effect was observed in the treatment with MG1363 (pExu:*gdf11*).

### Recombinant *L. lactis* (pExu:*gdf11*) strains regulated inflammatory markers of mucositis

**FIGURE 5:**
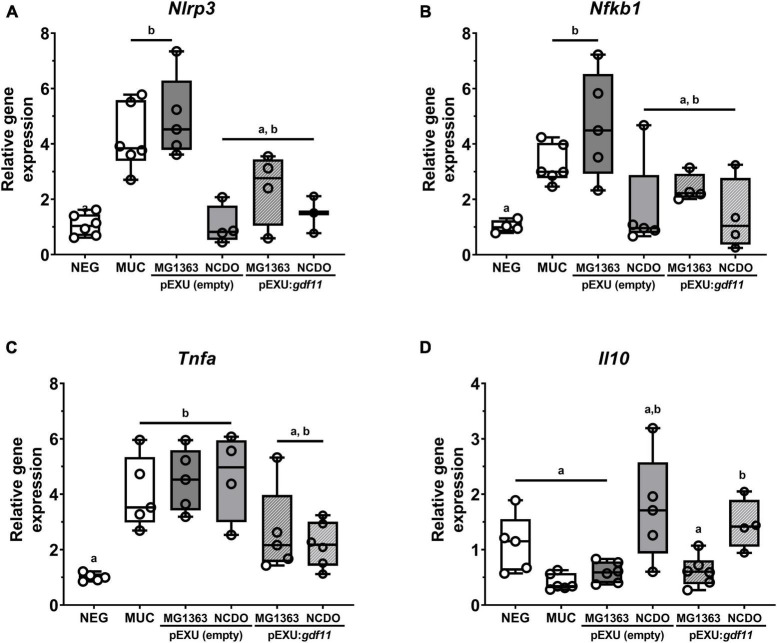
Immunomodulatory effect of *recombinant L. lactis* (pExu:*gdf11*) on inflammatory markers of mucositis (*n* = 3–5). **(A)** *Nlrp3*, **(B)** *Nfkb1* (p105), **(C)** *Tnf*, and **(D)** *Il10* following 2^ΔΔCt^ method of analysis. Different letters (a and b) indicate a significant difference (*p* < 0.05) among groups by one-way ANOVA and Tukey’s multiple comparison post-test.

To better understand the potential anti-inflammatory effect of our recombinant strains, we performed gene expression analysis of inflammatory markers. Figure 5 shows an increase in gene expression of pro-inflammatory genes *Nlrp3, Nfkb1*, and *Tnf* and no alteration of *Il10* transcript levels after 5-FU administration (MUC group) compared to the negative control. Treatment with both *L. lactis* NCDO2118 (pExu:*gdf11*) and *L. lactis* MG1363 (pExu:*gdf11*) showed a reduction in transcript levels of *Nlrp3, Nfkb1*, and *Tnfa*, demonstrating an immunomodulatory activity in the inflammatory process that can be attributed by rGDF11 delivered by *L. lactis* in mice with intestinal mucositis. We also observed an increase of *Il10* levels in the group that received *L. lactis* NCDO2118 (pExu:*gdf11*), demonstrating the potential immunomodulation of this recombinant strain.

## Discussion

Using microorganisms with probiotic properties as delivery vehicles of recombinant biomolecules emerges as a promising approach for treating inflammatory diseases (Carvalho et al., 2017b; Tavares et al., 2020). More recently, several studies have demonstrated the attenuating effects of recombinant targets delivered by LAB, mainly *L. lactis* strains in mouse models related to GIT inflammatory conditions such as UC and intestinal mucositis induced by 5-FU (Del Carmen et al., 2012; Souza et al., 2016; Barroso et al., 2021). In this context, *L. lactis* NZ9000 secreting recombinant *pancreatitis-associated protein* (PAP) could be associated with preserving intestinal villus and increasing Paneth cells’ granules in a mice model of intestinal mucositis induced by 5-FU (Carvalho et al., 2017a). *L. lactis* subsp. *cremoris* MG1363 carrying eukaryotic expression vector pValac:*il10* ameliorated UC in an experimental mice model, attenuating disease score and decreasing *Il6* levels (Zurita-Turk et al., 2014). Administration of *L. lactis* NCDO2118 secreting recombinant heat-shock protein 65 (HSP65) also prevented UC in mice by lowering histopathological score, decreased levels of pro-inflammatory cytokines IFN-γ, IL-6 e TNF, and increased levels of IL-10 (Gomes-Santos et al., 2017). On the other hand, following the administration of this recombinant strain in a model of arthritis, the oral treatment decreased levels of IL-17 and IFN-γ and prevented inflammatory infiltration and bone degeneration in the tissue (Gusmao-Silva et al., 2020).

*Lactococcus lactis* MG1363 and NCD02118 strains have been used as promising probiotic bacteria in gut inflammation context (Luerce et al., 2014; Alves et al., 2023). However, in addition to the fact that few studies have evaluated the effect of the recombinant form of these strains (Gomes-Santos et al., 2017; Fang et al., 2019), no studies explore the delivery potential of therapeutic molecules by these recombinant probiotics strains in intestinal mucositis. Thus, based on these findings, we sought to investigate the therapeutic potential of recombinant strains MG1363 and NCDO2118 in intestinal mucositis induced by 5-FU. Moreover, in the search for new therapeutical molecules, the potential anti-inflammatory role of GDF11 has only recently begun to be elucidated on different experimental models such as arthritis (Li et al., 2019), psoriasis (Wang et al., 2019), and UC (Wang et al., 2018) in mice, where it has demonstrated a suppression role in the pro-inflammatory NF-κB pathway and lowered expression levels of pro-inflammatory cytokines TNF, IL-1β, and IL-6 (Wang et al., 2018, 2019; Li et al., 2019).

Mucositis is a side-effect of 5-FU-chemotherapy that causes clinically relevant aspects such as weight loss. In addition, epithelial cell death, loss of the small intestine architecture, goblet cell degeneration, an increase of MPO activity, and expression of pro-inflammatory genes such as TNF, IL-6, and IFN-γ are reported (van Vliet et al., 2010; Dahlgren et al., 2021; Sougiannis et al., 2021). In this study, we observed an attenuating effect in the inflammatory parameters, but the administration of *L. lactis* (pExu:*gdf11*) did not prevent weight loss. Other studies that utilized the NCDO2118 strain in a mice model of UC demonstrated the prevention of this parameter. However, for intestinal mucositis induced by 5-FU, neither the administration of other probiotic recombinant strains *L. lactis* NZ9000 nor *L. delbrueckii* CIDCA 133 were able to prevent weight loss, demonstrating that this parameter could be experimental model specific (Luerce et al., 2014; Carvalho, 2016; Carvalho et al., 2017a; Barroso et al., 2021). Moreover, the villus shortening and crypt loss caused by 5-FU toxicity can be directly associated with the malabsorption of nutrients and might contribute to the weight loss observed (Yeung et al., 2015). In our study, we observed a loss of villus and crypts due to 5-FU damage, and treatment did restore these cells in the mucosa to a normal level, corroborating previous results found in mucositis treated with recombinant *L. delbrueckii* (pExu:*hsp65*) (Barroso et al., 2022).

Following the confirmation of the successful delivery of rGDF11 through gene expression analysis on the tissue, further investigation of the mucosal damage was carried out. Regarding the intestinal mucosa, goblet cells are essential in the secretion of mucin and other mucus components in the small intestine and thus, considered important protection of the epithelial surface (Pelaseyed et al., 2014). In our study, it was possible to observe a protective effect on goblet cell preservation caused by the administration of strain NCDO2118, demonstrating that this microorganism had an ameliorative effect on the intestinal mucosa. Additionally, this protective effect on goblet cells was sustained when recombinant strains carrying pExu:*gdf11* were administered. Similar results were found for other probiotic strains in 5-FU-induced mucositis (Yeung et al., 2015; de Jesus et al., 2019) and for recombinant *L. delbrueckii* (pExu:*hsp65*) administered in a 5-FU-induced mucositis model in mice, demonstrating the potential of this therapeutic approach toward this parameter (Barroso et al., 2021). The damage caused by 5-FU toxicity and the anti-inflammatory effects of recombinant *L. lactis* (pExu:*gdf11*) on the mucosa were assessed through a histopathological score. Results found in this study showed that administration of *L. lactis* NCDO2118 (pExu:*gdf11*) was able to decrease the score when compared to the MUC group. Similar results were found in studies that administered probiotic strains in 5-FU-induced mucositis, in which a protective effect on the intestinal mucosa was achieved and thus present a potential treatment alternative (Yeung et al., 2015; Barroso et al., 2022).

The inflammatory process on the mucosa leads to the recruitment of polymorphonuclear cells such as neutrophils to the inflammation site and thus, the neutrophilic infiltration in GIT can be assessed through the activity of the MPO enzyme (Hamouda et al., 2017). In this study, we observed that administration of strain *L. lactis* NCDO2118 (pExu:*gdf11*) decreased neutrophil activity on the tissue in comparison to the MUC group, suggesting an anti-inflammatory effect that could partly explain the ameliorating effect observed (Soares et al., 2011, 2013). In the study that utilized recombinant *L. lactis* secreting pancreatitis-associated protein I (PAP) in a mice model of mucositis, lower levels of MPO activity were also found and associated with the protective effect on the mucosa of this recombinant strain (Carvalho et al., 2017a). Similar results were also found in a study following the administration of *L. delbrueckii* (pExu:*hsp65*) in 5-FU-induced mucositis (Barroso et al., 2021).

5-fluorouracil damage leads to the liberation of ROS and pro-inflammatory cytokines in the intestinal mucosa, which are majorly driven by the activation of the NF-κB pathway. NF-κB is a multiprotein complex that plays a significant role in coordinating innate and adaptative immune responses, involved in inflammatory processes such as diseases of the GIT (Chang et al., 2012). The activation of NF-κB is reported in intestinal mucositis induced by 5-FU, leading to the production of pro-inflammatory cytokines TNF, IL-6, and IFN-γ and apoptosis signals that contribute to intestinal damage (Sougiannis et al., 2021). Moreover, the NF-κB pathway activates the NLRP3 inflammasome complex which is the main responsible for the maturation of pro-inflammatory cytokine IL-1β and is markedly present in mucositis induced by 5-FU (Soares et al., 2013; Wang et al., 2018).

The administration of *L. lactis* (pExu:*gdf11*) was able to decrease *Nlrp3, Nfkb1, Tnf* and increase *Il10* mRNA levels compared to MUC group. These results can corroborate previous results found for GDF11. In a study reporting the role of GDF11 in inhibiting the inflammatory process on mice skin similar to psoriasis, the attenuation of the disease could be attributed to blockage of NF-κB signalization pathway, being reported low levels of expression when rGDF11 was administered i.p. (Wang et al., 2019). Studies in arthritis and UC in mice demonstrated that GDF11 could inhibit TNF levels in other experimental models, and other studies suggest that upregulation of *Tnf* is linked to epithelial regeneration and thus could be a mechanism of action of probiotic bacteria in promoting intestinal barrier restoration and immunity (Pagnini et al., 2010; Giorgetti et al., 2015; Wang et al., 2018; Li et al., 2019; Barroso et al., 2022). Regarding IBDs, GDF11 was able to ameliorate UC in a mice model through inhibition of NLRP3 inflammasome complex, the main responsible for the maturation of pro-inflammatory cytokine IL-1β and markedly present in mucositis induced by 5-FU (Soares et al., 2013; Wang et al., 2018). In the present study, although the levels of IL-1β were not evaluated, we found lower expression levels of *Nlrp3* in the groups treated with *L. lactis* (pExu:*gdf11*), demonstrating immunomodulation of this multiprotein complex, and corroborating similar results previously found in Ulcerative Colitis disease model and neointimal hyperplasia model utilizing GDF11 (Wang et al., 2018; Li et al., 2022).

Moreover, the balance of pro and anti-inflammatory molecules is crucial in directing an acute process toward the resolution of inflammation and minimizing tissue damage. Cytokine IL-10 has a significant effect on the TGI since knockout of *Il10* or its receptor leads to colitis predisposition in experimental models as well as reduction of circulating levels of IL-10 were found in mice with mucositis, and increased levels were crucial in attenuating mucositis in mice treated with *Lactobacillus acidophilus* (Justino et al., 2015; Jain et al., 2017). Notably, IL-10 can also modulate neutrophil metabolism toward angiogenesis and tissue healing (Bazzoni et al., 2010). Taken together, we suggest that the administration of these recombinant pExu:*gdf11* strains were able to modulate inflammation through interaction with the NF-κB pathway, which led to a suppression of the inflammasome complex activation and also to increased levels of IL-10 cytokine production and this immunomodulation can help to explain the ameliorative effect in mucositis. Moreover, further investigation into GDF11’s anti-inflammatory role such as other anti-inflammatory pathways should be assessed in the future.

## Conclusion

Our results showed that recombinant *L. lactis* (pExu:*gdf11*) strains were able to partially ameliorate the intestinal damage of mucositis by preventing infiltration of neutrophils, preserving goblet cells count and intestinal mucosa architecture. These results could be partially explained by an immunomodulation effect through the NF-κB pathway and increased *Il10* expression levels. Hence, we believe that this approach offers a promising alternative to be explored in the treatment of intestinal mucositis. Nevertheless, we emphasize that more studies should be performed to better elucidate the mechanisms of *Gdf11* immunomodulation, the effect on the intestinal permeability proteins, and the impact on microbiome modulation.

## Data availability statement

The original contributions presented in this study are included in the article/supplementary material, further inquiries can be directed to the corresponding author.

## Ethics statement

This animal study was reviewed and approved by the Local Animal Experimental Ethics Committee of Federal University of Minas Gerais (CEUA-UFMG).

## Author contributions

MA, RC, and VA: conceptualization. MA, AF, TS, FB, RS, GG, GC, RA, JL, and PM-A: methodology. MA, LJ, and ÊF: formal analysis and investigation. MA: original draft writing preparation. LJ, PM-A, RC, J-MC, and VA: writing—review and editing. All authors contributed to the article and approved the submitted version.
